# Giant Switchable Remanent Polarization and Photocurrent in Ferroelectric Thin Film Photomemristor for In Situ Training

**DOI:** 10.1002/advs.202517077

**Published:** 2026-02-27

**Authors:** Zhen Zhao, Chengze Sun, Zhijin Duo, Yue Hou, Zhanfeng Wang, Jikang Xu, Ziye Li, Fu Wang, Pengfei Li, Ying Liu, Yongqing Jia, Kangbo Zhao, Jia Wu, Biao Yang, Weifeng Zhang, Weidong Sun, Jiacheng Wang, Jinxia Liu, Junfeng Yu, Xiang Ying, Jianxin Guo, Xiaobing Yan

**Affiliations:** ^1^ Key Laboratory of Optic‐Electronic Information Materials of Hebei Province, School of Life Sciences, Institute of Life Science and Green Development, Key Laboratory of Brain‐Like Neuromorphic Devices and Systems of Hebei Province, College of Physics Science and Technology Hebei University Baoding China; ^2^ College of Electronic and Information Engineering Hebei University Baoding China

**Keywords:** ferroelectric thin films, in situ training, photocurrent, photomemristor, remanent polarization

## Abstract

Ferroelectric thin film photomemristors with a stable and tunable short‐circuit current offer a promising solution for developing machine vision systems with in situ training. However, previously reported devices exhibit inadequate remanent polarization and limited short‐circuit currents, posing significant challenges to the efficient implementation of in situ training in complex environments. In this work, we realized the in situ training in a machine vision system through the Pt/SrRuO_3_/PbZr_0.4_Ti_0.6_O_3_/SrRuO_3_/SrTiO_3_ device. Induced by the bottom electrode‐induced stress, the device shows a large remanent polarization of 94 µC cm^−2^ and an ultra‐high short‐circuit current of 183.35 nA. Furthermore, the stable and tunable multilevel photocurrents of this device enable image edge processing and in situ multifunctional neural signal training for miniature vehicles in machine vision systems. This work demonstrates the immense potential of ultra‐high‐performance ferroelectric thin film photomemristors for advancing machine vision systems research.

## Graphical abstract

The device exhibits excellent ferroelectric performance and significant photoelectric response. It can adapt to light stimuli of varying power and wavelengths while achieving a stable and tunable photovoltaic response state. Moreover, the device can utilize photoconductive states of different directions and magnitudes to achieve pattern classification and in situ training in the machine vision system.

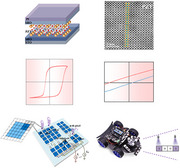

## 1 Introduction

Machine vision systems can capture images through hardware devices such as cameras and sensors, then analyze and process these images using algorithms to achieve automated functions such as recognition, detection, and measurement [1, 2]. However, as machine vision systems confront dual pressures from dynamic and complex operational environments and increasing demands for real‐time efficiency, the inherent limitations of the von Neumann architecture become critically evident, manifesting as data transfer bottlenecks leading to latency‐induced performance degradation, computing‐memory bandwidth mismatch resulting in parallel processing inefficiencies, and redundant data flows contributing to increased power consumption [3, 4]. Studies reveal that novel architecture hardware for computing‐memory convergence with neuromorphic computing and multimodal fusion features is expected to become a solid platform for machine vision systems in the post‐Moore Era [5, 6]. Moreover, novel photoelectronic computing‐memory convergence devices are ideal for breaking through the bottlenecks of the von Neumann architecture with their efficient parallel processing, low power consumption, high temporal resolution, and compact integrated design, and offer significant advantages when applied in machine vision systems [7, 8]. To date, numerous materials have been widely reported for use in photoelectronic computing‐memory convergence devices capable of integrating optoelectronic conversion and data processing, such as ferroelectric materials [9, 10], phase change materials [11, 12], perovskite materials [13, 14], low‐dimensional materials [15, 16], organic photoelectronic materials [17, 18], and sulfides [19, 20], which find broad applications in machine vision systems and other high‐efficiency computing and storage fields. However, many ionic conductive materials‐based photoelectronic devices suffer from poor environmental stability, and material layer compatibility issues [21]. Their photovoltaic response mainly relies on ion migration within the materials to achieve tunable light response, which prevents the devices from integrated and operating stably in complex practical environments [22].

Interestingly, ferroelectric photovoltaic device is able to directly perceive, memory, and compute information using light, providing a new hardware solution for breaking through the energy efficiency and speed bottlenecks of traditional computing architectures. Photodetectors incorporating ferroelectric materials have recently attracted interest because of their scalability, self‐terminated surfaces, and low defect densities, which provide an additional degree of freedom to achieve more possibilities in optoelectronics application [23, 24]. Moreover, ferroelectric photovoltaic devices utilizing materials integrating ferroelectricity, photovoltaic effects, and memristive characteristics can mimic the synaptic functions of biological visual systems, rendering them well‐suited for machine vision systems requiring rapid processing and environmental adaptability [25, 26]. In neuromorphic devices, the short‐circuit current (*I*_sc_) generated by ferroelectric photovoltaic effects directly corresponds to synaptic weights [25]. When light intensity or polarization state changes, the adjustment of amplitude or direction of this *I*_sc_ reflects synaptic weight updates; such a mechanism obviates the need for additional electrical pulse writing, thereby significantly reducing energy consumption [27]. The *I*_sc_’s direct responsiveness to optical signals enables the construction of light‐controlled neuromorphic systems, whose light‐driven capability can substantially enhance information processing speed and accuracy in applications such as visual processing [28]. Furthermore, the *I*_sc_ can be finely regulated via electric fields to achieve multilevel synaptic weight storage, with its multistate characteristics providing a hardware foundation for training complex neural networks [29]. Unfortunately, the *I*_sc_ intensity of reported ferroelectric photovoltaic devices remains relatively limited, which represents a critical bottleneck restricting their performance [30]. For ferroelectric photovoltaic devices, this limited *I*_sc_ renders optical signals difficult to store and accurately read out, thereby significantly constraining the performance boundaries of information storage [30], and posing substantial challenges for effective machine vision applications in complex environments [31, 32]. Therefore, how to develop ferroelectric photovoltaic devices with higher performance and more functions has become an urgent issue to be addressed. Current research on ferroelectric photovoltaic devices primarily focuses on exploring new materials and optimizing material properties, with a lack of innovation in device structure [33]. Furthermore, the coupling between the polarization of ferroelectric domains and film strain is crucial for enhancing the ferroelectric remanent polarization (*P*_r_) and optical responsivity [34]. By optimizing the mechanical stress applied to the ferroelectric layer, ferroelectric photovoltaic devices can exhibit improved ferroelectric properties and enhanced *I*_sc_ [35]. In the applications of multiple photocurrent states, the enhanced photocurrent exhibits remarkable distinguishability, which can simplify the design of circuit modules and enable them to perform more complex visual processing tasks in machine vision systems [36].

**FIGURE 1:**
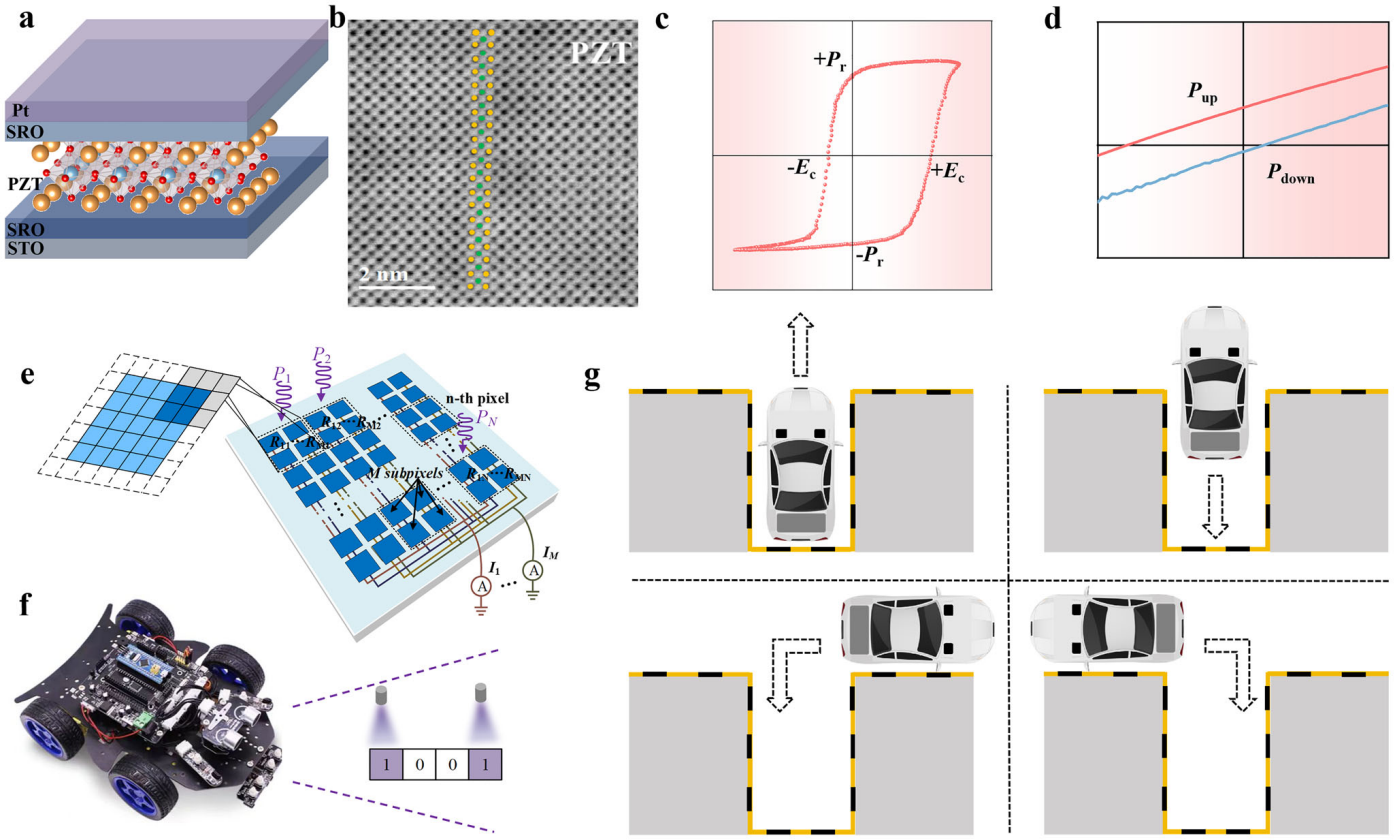
Ultrahigh‐photoresponsive ferroelectric thin film photomemristor for image recognition and in situ training. (a) Schematic of the device architecture. (b) Dark‐field STEM images focused on the PZT atomic arrangement of the unit cell. (c) *P–E* hysteresis loop. (d) Photovoltaic response of the Pt/SRO/PZT/SRO/STO device under illumination. (e) Schematic of the ferroelectric memristor network. (f) Schematic of the smart car system and its working principle. (g) Experimental demonstration of the smart car's reversing action.

In this work, we propose an enhanced strategy to develop machine vision systems by employing electrode strain engineering to optimize the ferroelectric properties and photovoltaic response of ferroelectric thin film photomemristor. As illustrated in Figure 1a, the Pt/SrRuO_3_ (SRO)/PbZr_0.4_Ti_0.6_O_3_ (PZT)/SrRuO_3_ (SRO)/SrTiO_3_ (STO)‐structured device benefits from using SRO as the electrode layer, which ensures the epitaxial quality of the PZT film. This design aims to maintain the substrate (STO) and ferroelectric layer (PZT) unchanged, and explore the role of the electrode interface in ferroelectric properties and photoresponse by systematically changing the electrode material (Figure S1). The compressive stress exerted by SRO electrode promotes superior epitaxial growth of the PZT film (Figure 1b). The device demonstrates outstanding ferroelectric characteristics (Figure 1c), achieving a maximum remanent polarization (*P*_r_) of 94 µC cm^−2^. Under 405 nm blue‐violet illumination, the device exhibits an *I*_sc_ of 183.35 nA (Figure 1d). Furthermore, the continuous tunability of its photoconductive synaptic behavior provides a critical foundation for developing neuromorphic computing architectures. The ferroelectric thin film photomemristor‐based network (Figure 1e) accomplishes pattern classification through multi‐level *I*_sc_ responses under distinct polarization orientations. Notably, we leveraged the device's photovoltaic synaptic characteristics to enable in situ training for controlling multifunctional movements of miniature vehicles within machine vision systems (Figure 1f,g). This research highlights promising prospects for constructing high‐performance self‐powered machine vision systems capable of operating in complex environments.

## 2 Results

### 2.1 High Quality Ferroelectric Thin Film Photomemristor

**FIGURE 2:**
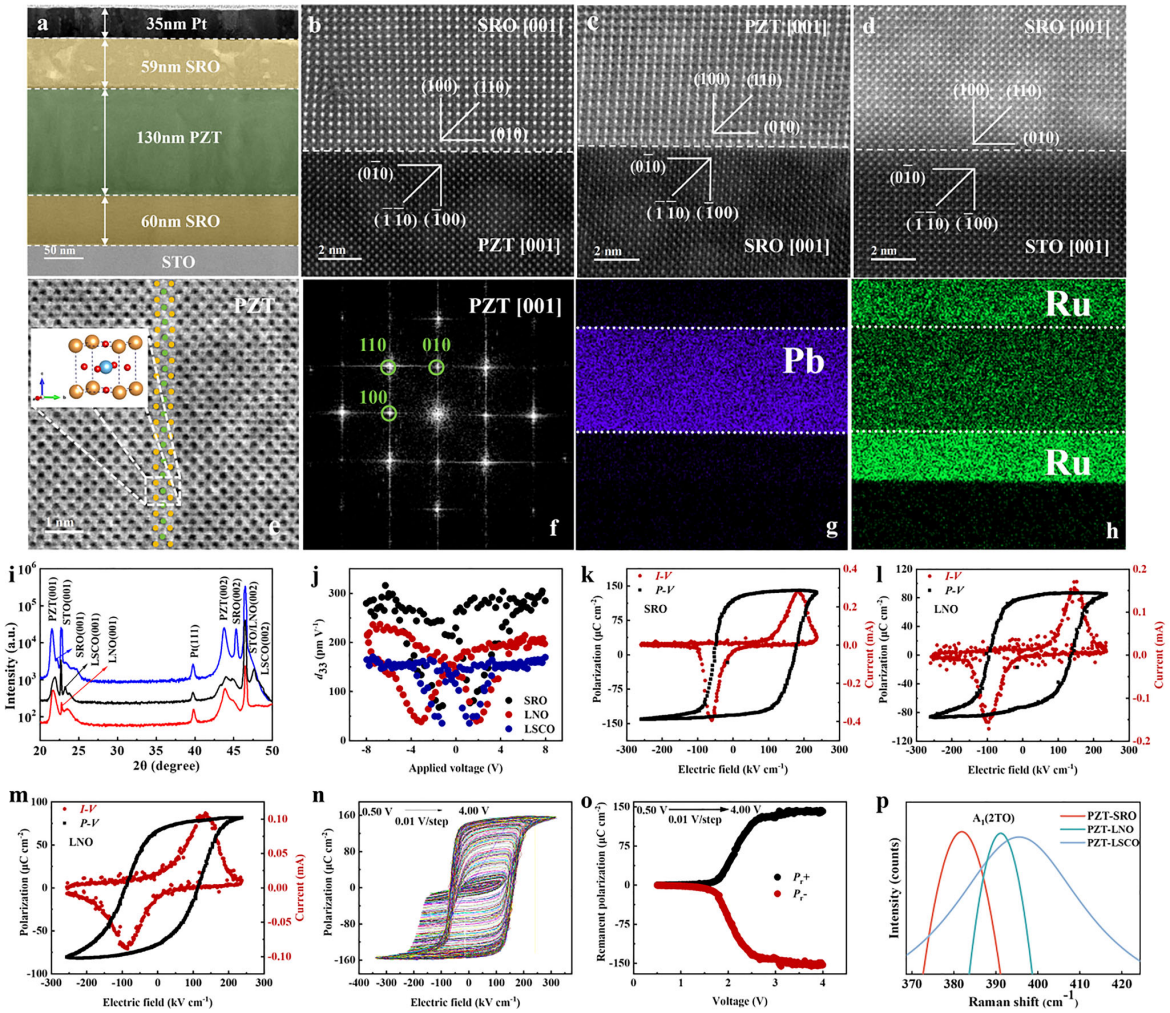
Characterization of the ferroelectric thin film photomemristor. (a) Low‐magnification STEM image of the device. (b–d) High‐angle annular dark‐field STEM images of (b) the SRO/PZT interface, (c) PZT/SRO interface, and (d) SRO/STO interface. (e) Bright‐field STEM image focused on the PZT atomic arrangement of the unit cell. (f) FFT analysis of a selected region in the PZT layer. (g) Pb elemental mapping in the PZT layer. (h) Ru elemental mapping in the SRO layer. (i) XRD patterns of Pt/ (SRO/LNO/LSCO)/ PZT/ (SRO/LNO/LSCO)/ STO devices. (j) Piezoelectric coefficient measurements of Pt/ (SRO/LNO/LSCO)/ PZT/ (SRO/LNO/LSCO)/ STO devices. (k–m) *P–E* hysteresis loops for devices with (k) SRO, (l) LNO, and (m) LSCO electrodes. (n) *P–E* hysteresis loops of the device after applying different voltage‐writing pulses. (o) Statistical analysis of *P*_r_ for devices with different voltage‐writing pulses. (p) Localized magnification of phonon mode vibrations A_1_(2TO) for three different structures.

The microstructure of the device heterojunction films were characterized using scanning transmission electron microscopy (STEM). Figure 2a shows a low‐magnification STEM image of the device, from which the thicknesses of the Pt, SRO (top electrode), PZT, and SRO (bottom electrode) films were determined to be approximately 35, 59, 130, and 60 nm, respectively. By zooming in on the bracketed interface regions in Figure 2a, atomic‐resolution high‐angle annular dark‐field STEM images were obtained for the SRO (top electrode)/PZT interface (Figure 2b), PZT/SRO (bottom electrode) interface (Figure 2c), and SRO (bottom electrode)/STO interface (Figure 2d). The PZT and SRO layers exhibit epitaxial alignment with the STO substrate. In the high‐magnification bright‐field STEM image of PZT (Figure 2e), the atomic structure of PZT is clearly observable, with its specific atomic structure model and corresponding crystal planes labeled in different colors: orange and green represent lead and titanium (zirconium) atoms, respectively. Fast Fourier Transform (FFT) analyses performed on selected regions of PZT, SRO (I/II) (where I = top electrode, II = bottom electrode), and STO (Figure 2f; Figure S2a–c) confirm the successful growth of high‐quality SRO/PZT/SRO/STO epitaxial heterojunction films. Elemental distribution maps of the device (Figure 2g,h; Figure S2d–f) further validate the structural integrity. These results are critical for understanding the origins of PZT's large polarization and high *I*_sc_, while also providing a foundation for designing ferroelectric devices based on PZT.

To characterize the orientation and crystallization quality of the three structured thin films, X‐ray diffraction (XRD) was used to collect patterns for samples with three different electrodes grown on STO substrates (Figure 2i). In the XRD scanning range of 20–50°, the PZT thin films in all three structures, along with the electrode materials (SRO, LaNiO_3_ (LNO), La_0.5_Sr_0.5_CoO_3_ (LSCO)), exhibit only (00*l*) orientation without additional diffraction peaks, indicating the growth of highly oriented single‐crystalline thin films. It can be observed that the peak of the PZT device on the SRO electrode is the sharpest, indicating that this structure has the best epitaxial and crystalline quality on the STO substrate. The piezoelectric constant *d*_33_, representing electric displacement per unit mechanical stress along the polarization direction, was measured [37]. As shown in Figure 2j, the device with an SRO electrode exhibited the highest *d*_33_ value of 316 pm V^−1^. Ferroelectric hysteresis loops were measured using the capacitance coupling method with a driving voltage of 3 V and frequency of 1 kHz (Figure 2k–m), all of which showed fully saturated characteristics. PZT‐SRO displayed the highest *P*_r_ and higher *E*_c_, which can be attributed to the superior lattice constant matching between PZT and SRO reducing lattice relaxation and enhancing tetragonality, thereby increasing *P*_r_ [38]. In order to provide clear evidence of intrinsic ferroelectricity and obtain accurate *P*_r_ and *E*_c_, we conducted positive up negative down (PUND) pulse measurements (Figure S3a), and the true *P*_r_ of PZT devices extracted from PUND was 94 µC cm^−2^. The extracted *P*_r_ and *E*_c_ values at different frequencies have small fluctuations, indicating good stability of the device (Figure S3b). In order to obtain the piezoresponse force microscopy (PFM) hysteresis loop and domain diagram of the device, we used PFM probes to measure PFM within the device region. Figure S3c provides an out of plane PFM phase domain diagram, clearly showing the uniform and stable polarization domain structure within the device region; The local piezoelectric hysteresis loop provided at the same time (Figure S3d) directly proves the polarization reversal behavior at the nanoscale, and its local coercive voltage is similar to the macroscopic electrical measurement results. In addition, in Figure S4, anti‐fatigue characteristics tests were conducted on devices based on SRO, LNO, and LSCO bottom electrodes. Compared with LNO and LSCO bottom electrode devices, SRO‐based bottom electrode device still exhibits stability after cycling through 10^10^ *P‐E* hysteresis loops, indicating that SRO‐based bottom electrode device has the best stability. As shown in Figure S5. Three types of bottom electrode devices were tested for leakage current at a constant input voltage of 3 V. Among them, the leakage current of the SRO bottom electrode device was the smallest, <2 × 10^−10^ A, indicating that the SRO bottom electrode device has the best thin‐film quality. And the SRO bottom electrode devices exhibit stable and repeatable temperature‐dependent characteristics after being subjected to temperatures up to 300°C (Figure S6).

The polarization reversal process begins with applying a low electric field amplitude of 0.5 V, which may fail to fully switch all ferroelectric domains, resulting in a small and potentially unsaturated hysteresis loop. As depicted in Figure 2n, the applied electric field amplitude is then gradually increased in 0.01 V steps, with corresponding hysteresis loops recorded at each amplitude level. At a higher electric field amplitude of 4 V, the hysteresis loop fully saturates, exhibiting maximum *P*_r_ and coercive field. As shown in Figure 2o, modulating the applied electric field pulse amplitude induces corresponding changes in *P*_r_, reflecting the degree of domain switching within the material during pulsing. As shown in Figure S7, applying electric field pulse width also induces corresponding changes in *P*_r_. To further validate whether performance variations among the three structures originate from stress effects, Raman spectroscopy measurements were performed on PZT layers of the three structures. Figure 2p shows magnified A_1_(2TO) phonon mode vibration spectra for the three samples. The Raman peak shift is linearly proportional to the applied stress. As illustrated, the A_1_(2TO) Raman shifts for PZT‐SRO, PZT‐LNO, and PZT‐LSCO decrease in the order of PZT‐SRO > PZT‐LNO > PZT‐LSCO. Using established stress‐calibration Equation (1) [39, 40],

(1) $$\omega \left(\sigma \right)=\omega \left(0\right)-\frac{\partial \omega }{\partial \sigma }\sigma$$

where *ω*(0) is the frequency of the A_1_(2TO) phonon mode under stress‐free conditions, and *ω*(σ) is proportional to the pressure σ. All devices exhibit compressive stress, with calculated values of 2.41 GPa (PZT‐SRO), 3.12 GPa (PZT‐LNO), and 3.56 GPa (PZT‐LSCO), respectively. Notably, the PZT‐LSCO device displays the lowest A_1_(2TO) peak position, indicating more pronounced stress impacts on its performance—consistent with the ferroelectric property trends. Altering the device's electrode material influences film stress: the PZT‐SRO device's superior lattice matching reduces residual stress, as deduced from the formula. This further confirms that lattice mismatch‐induced dislocations generate internal stress fields, and lower lattice mismatch facilitates stress relaxation, thereby optimizing film structure and performance [41]. Comparison with previously reported PZT‐based ferroelectric devices shows that *P*_r_ in this work is the largest (Table S1).

### 2.2 High Photovoltaic Response Ferroelectric Thin Film Photomemristor

**FIGURE 3:**
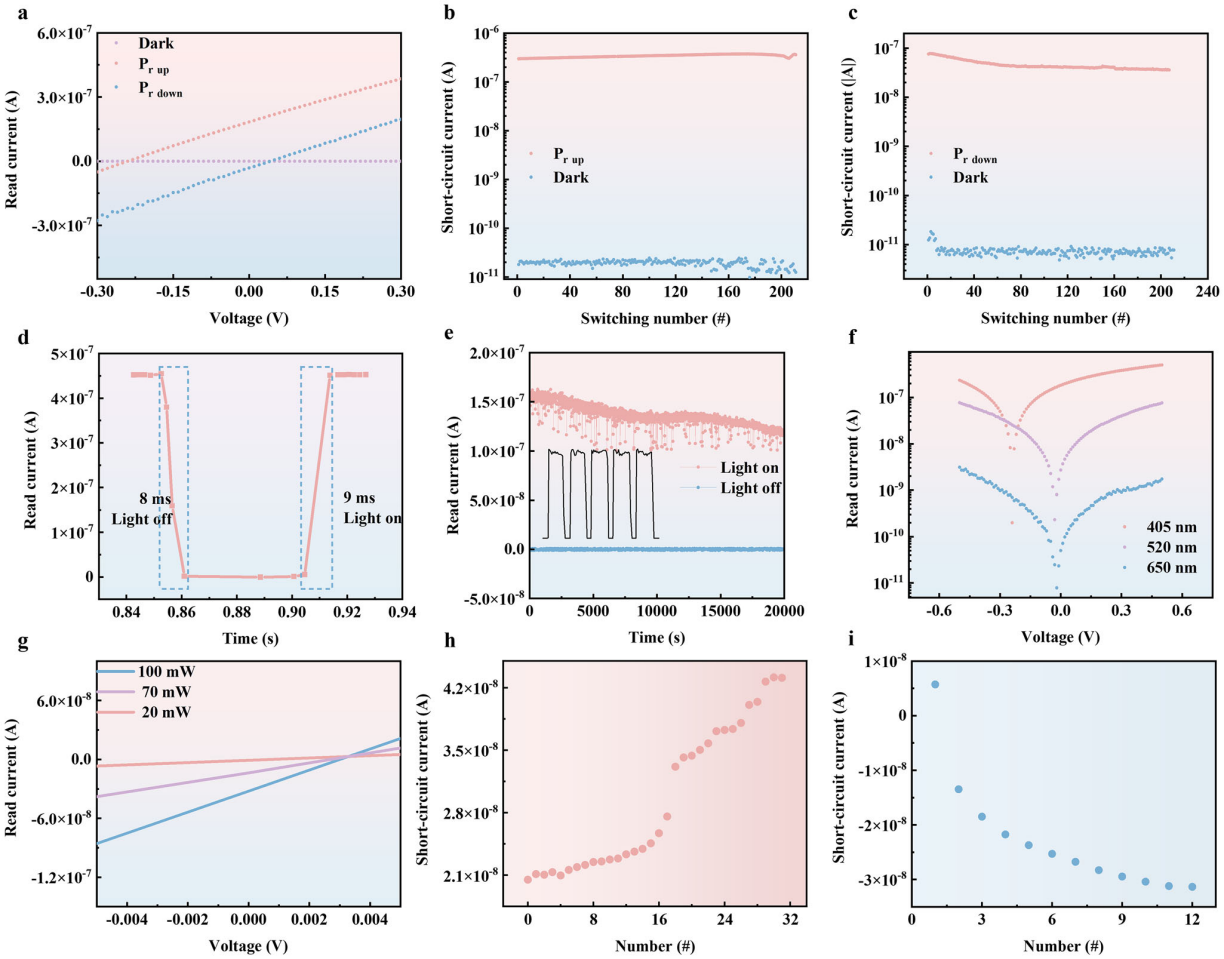
Photovoltaic behavior of Pt/SRO/PZT/SRO/STO devices. (a) *I–V* characteristics of the device in dark and illuminated states under *P*_up_ and *P*_down_ states, and statistical analysis of (b) *I*_sc_ for *P*_up_ and (c) *P*_down_ states, with corresponding *I–V* curves under light/dark switching. (d) Response speed of the device to light pulse modulation. (e) Retention characteristics of optical pulse‐induced resistance states in the *P*_up_ state. (f) *I–V* curves of the downward‐polarized device under light sources of different wavelengths. (g) *I–V* curves of the upward‐polarized device under light sources of different photon energies. (h,i) Polarization state modulates the variation trends of *I*_sc_ in (h) *P*_down_ and (i) *P*_up_ states.

Unlike conventional photoelectronic devices, ferroelectric photomemristor can generate an *I*_sc_ even in the absence of an external electric field, leading to distinctive *I–V* characteristics [42]. The *I–V* characteristics of no polarization, *P*_down_, and *P*_up_ states were studied under visible light illumination with a wavelength of 405 nm and an intensity of 100 mW cm^−2^, as shown in Figure 3a. The *I‐V* curve of the no polarization sample shows a negligible photovoltaic response. However, photovoltage and *I*_sc_ can be achieved in the poled samples. Similar to the *I*_sc_ responses, the *I*_sc_ and photovoltage can be reversed in the opposite direction after reversing the polarization direction. In addition, the *I‐V* curves of *P*_down_ and *P*_up_ states exhibit a slight asymmetrical state in terms of the absolute value of the difference in open‐circuit voltage (*V*_oc_) and *I*_sc_, as shown in Figure 3a. The *V*_oc_ and *I*_sc_ values of the *P*_up_ poled state are larger than those of *P*_down_ state, which is highly likely due to the asymmetric defect distribution inside the ferroelectric thin film. In addition, Schottky barrier modulation can effectively explain the polarization controlled switchable optical response in Pt/SRO/PZT/SRO/STO device (Figure S8). Comparison with previously reported ferroelectric photovoltaic devices shows that short‐circuit current density (*J*_sc_) in this work is the largest (Table S2). When switching between 405 nm light and dark conditions, testing the *I–V* curve and recording the device's *I*_sc_ reveals that in the *P*_up_ state, Figure 3b, the *I*_sc_ can stably maintain more than 200 cycles of switching between 10^−7^ A and 10^−11^ A. Similarly, as shown in Figure 3c, in the *P*_down_ state, the device can stably maintain more than 200 cycles of switching between 10^−8^ A and 10^−11^ A. The response speed is determined by how quickly the *I*_sc_ can rise or decay when the illumination is turned on or off, respectively. As shown in Figure 3d, the *I*_sc_ takes 8 ms to decrease from 90% to 10% of its maximum value when the light is turned off. It takes 9 ms for the *I*_sc_ to rise from 10% to 90% of its maximum value when the light is turned on. As shown in Figure 3e, a photovoltaic current switching cycle test over 20 000 s evaluates the endurance of a photomemristor's current generation under prolonged and repeated switching between illumination and dark conditions. This test is particularly relevant for assessing the long‐term reliability of devices in real‐world applications.

The *I‐V* response of the ferroelectric photomemristor under different wavelength illumination is an important characteristic that reveals how this device responds to varying light conditions. As shown in Figure 3f, the photovoltaic response of the device was tested under laser light sources with wavelengths of 405, 520, and 650 nm. Results show that under the 405 nm light source, the device exhibits the highest photovoltaic response, including maximum *I*_sc_ and *V*_oc_. As shown in Figure 3g, the photovoltaic response of the device was tested using 405 nm light sources with power levels of 20, 70, and 100 mW cm^−2^. Results indicate that the device exhibits the highest photovoltaic response when tested with the 100mW cm^−2^ laser. The variation trend of *I*_sc_ with polarization state switching in ferroelectric photomemristor is an important aspect that reflects the interplay between ferroelectric properties and *I*_sc_ generation. As shown in Figure 3h, *I*_sc_ decreases gradually from 2 × 10^−8^ A to 4.5 × 10^−8^ A with increasing the number of positive pulses, a manifestation of long‐term potentiation behavior. By contrast, as shown in Figure 3i, *I*_sc_ increases from 5 × 10^−9^ A to −3 × 10^−8^ A under negative pulse stimulation, indicating long‐term depression behavior. Similarly, as shown in Figure S9, *I–V* curves and statistical analysis of *V*_oc_ of the device in *P*_down_ state. Interestingly, the device utilizes *I*_sc_ to simulate Morse code characters and American standard code for information interchange (ASCII) numerical digits (Figure S10).

### 2.3 Implementations of Pattern Classification of Ferroelectric Photomemristor Network

**FIGURE 4:**
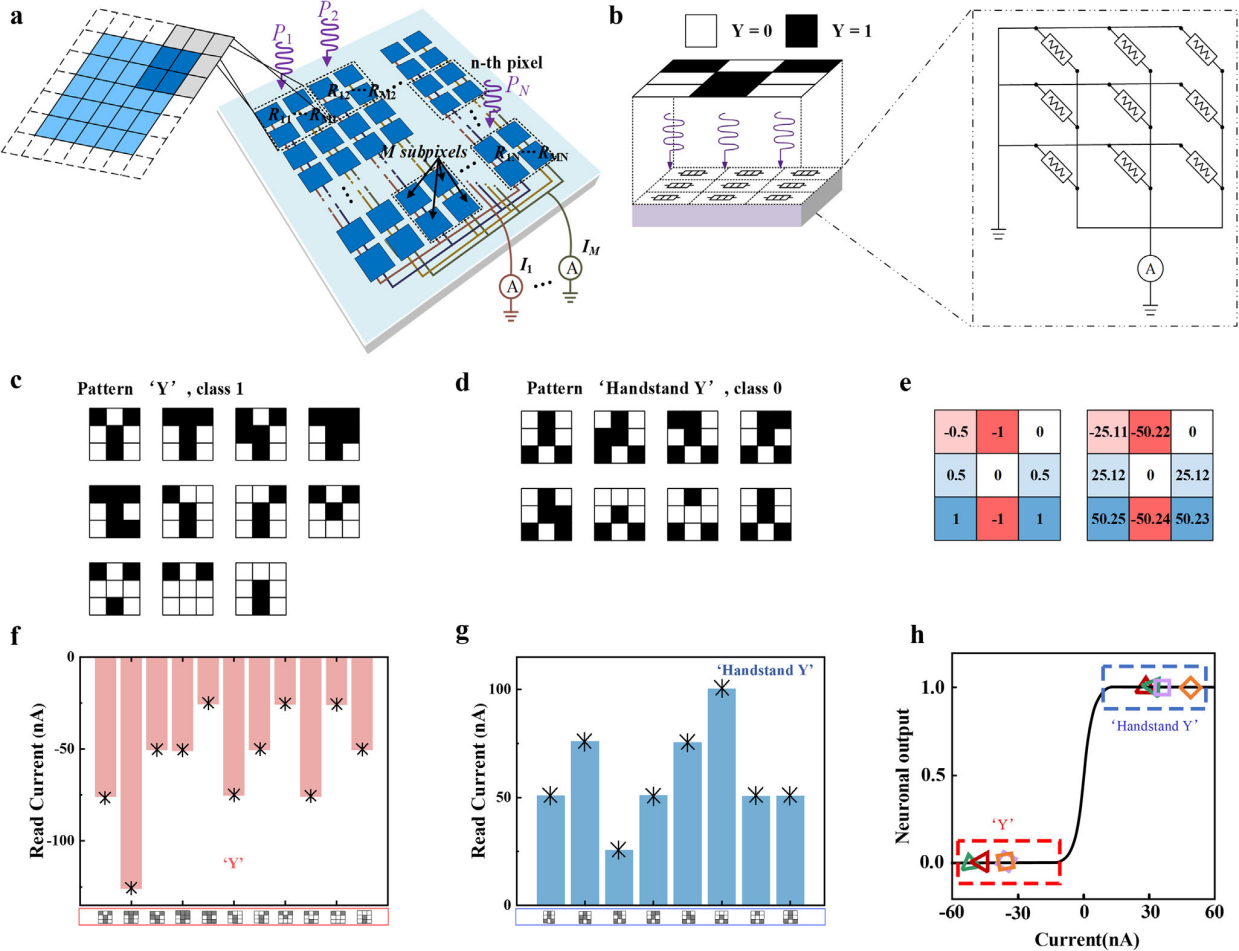
Pattern classification implementation. (a) Architecture of the ferroelectric thin film photomemristor network. (b) Schematic of the 1 × 9 ferroelectric photomemristor network, illustrating the operational principle and circuit structure. Pattern sets representing the characters (c) “Y” and (d) “⅄”, along with their noise‐augmented variants, are used for training and testing. (e) Theoretical dimensionless weights and theoretical photoresponsivities scaled from dimensionless weights are shown. The theoretical value of the output current during the presentation of (f) “Y” and (g) “⅄” input modes is denoted by asterisks. (h) The neuron output is obtained by inputting the output current into an *S‐*shaped function.

After demonstrating the switchable photoresponse of the ferroelectric photomemristor and understanding its physical mechanism, it becomes meaningful to explore hardware implementations that utilize a ferroelectric photomemristor network for simultaneous image sensing and processing. As shown in Figure 4a, the network is composed of *N* pixels, each divided into *M* subpixels. *N* depends on the image size, i.e., *N* = *H* × *W*, where *H* and *W* represent the height and width of the image, respectively. The *N* pixels are arranged in an *H* × *W* array to accommodate the image. The *M* subpixels are also arranged in a two‐dimensional array to save area overhead. Each subpixel corresponds to one device, which has a subpixel index (*m* = 1, 2, …, *M*) and a pixel index (*n* = 1, 2, …, *N*); the devices with the same subpixel index *m* are connected in parallel. Through this architecture, the ferroelectric photomemristor network is capable of performing efficient sensing, storage, and computing operations: under short‐circuit and illuminated conditions, during the light‐sensing process, each individual device performs a multiplication between the incident light power and its photoresponse; meanwhile, according to Kirchhoff's law, the *I*_sc_ generated by the *N* devices with the same subpixel index *m* is summed together. The expression for the output current Im is given by Equation (2):

(2) $$I_{m}=\sum_{n=1}^{N}R_{\mathrm{mn}}P_{n}$$

here, *R_mn_* denotes the photoresponse of the device at the *n*th pixel and the *m*th subpixel (simply referred to as the device at position (*m*,*n*)), and *P*_n_ represents the input light power at the *n*th pixel. In the experiment, two sets of patterns were classified, a task that can be implemented using a single‐layer perceptron comprising 9 input neurons and 1 output neuron. This single‐layer perceptron is hardware‐realized using a 1 × 9 (i.e., M = 1 and N = 9) ferroelectric photomemristor network (as shown in Figure 4b). As illustrated in Figure 4c,d, the patterns representing the letter “Y” and its inverted form “⅄”, along with their noisy variants, serve as both the training set and the test set. Each pattern consists of 3 × 3 = 9 pixels, with the pixel values defined as 1 for black and 0 for white.

When the ferroelectric photomemristor network is illuminated, a pixel value of 1 (or 0) indicates that 7.85 µW of light is applied (or removed) to the corresponding device at that position. Subsequently, an operation is performed between the input image and the photoresponse matrix, and the network produces an output current I_1_, which is then fed into an *S‐*type activation function to generate the neuron's output. The output current I_1_ is only on the order of a few nanoamperes, so first amplified by a circuit before being input into the *S‐*type activation function. Here, the *S‐*type activation function is implemented in software, and the training is also conducted in software—a method referred to as offline training. Then, the computed weight matrix is written into the ferroelectric photomemristor network. Figure 4e shows the computed weight matrix, while Figure 4f,g displays the output current I_1_ for different input patterns. When the light input belongs to the “Y” class, the output current is consistently negative, whereas when the light input belongs to the “⅄” class, the output current is consistently positive. Furthermore, the measured output current agrees well with the theoretical calculations. Figure 4h shows the neuron output obtained from the output current I_1_. For light inputs belonging to the “Y” class, the neuron outputs are close to 1, while for those belonging to the “⅄” class, the outputs are near 0, indicating that all patterns are correctly classified. Therefore, for this simple binary classification task, the accuracy is 100%.

### 2.4 In Situ Training of Multifunctional Signal Processing

**FIGURE 5:**
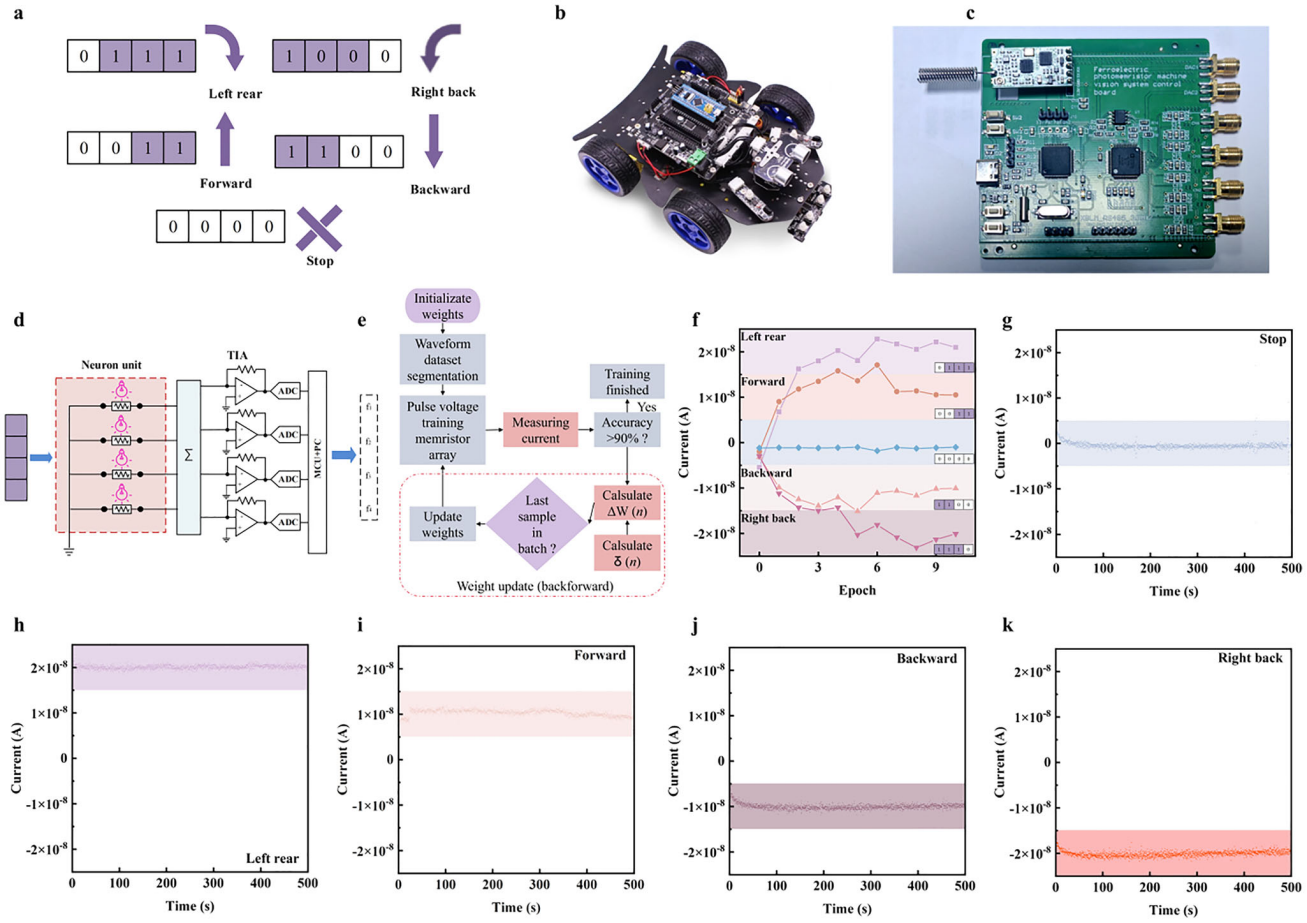
In situ training of the ferroelectric photomemristor network as an autonomous vehicle prototype “vision system” and its trained performance. (a) Five action markers are used for training and testing. (b) Complete vehicle photograph. (c) Schematic of a neuron unit comprising an amplification circuit and a comparison circuit. (d) Schematic and circuit diagrams of the system. (e) Flowchart for in situ training of the ferroelectric photomemristor network. (f) Training process of the ferroelectric photomemristor network. (g–k) *I*_outs_ of the ferroelectric photomemristor network during execution of different actions.

High‐performance ferroelectric photomemristor enables the construction of ferroelectric photomemristor network with visual recognition capabilities. Such a network can serve as the “visual system” of an autonomous vehicle. Figure S11 clarifies how steering commands are physically received in the autonomous driving demo. As shown in Figure 5a, the system is trained using four 4 × 1 pixel reverse parking signs that represent five commands: “left rear”, “forward”, “stop”, “backward”, and “right back”. These reverse parking signs are generated by a 4 × 1 laser array, with each laser aimed at one ferroelectric photomemristor. The pixel values “1” and “0” correspond to light intensities of 100 and 0 mW cm^−2^, respectively. The four ferroelectric photomemristors are connected in parallel to form a 4 × 1 ferroelectric photomemristor network. For each pixel, the corresponding device produces an *I*_sc_ by multiplying its photoresponsivity by the light power in that pixel, and the total *I*_sc_ output from all devices is summed according to Kirchhoff's law. The network is then trained to recognize the reverse parking sign. The network's recognition result is directly sent to the vehicle's motion system (Figure 5b,c) to control its direction. The output current is given by the following Equation (3):

(3) $$I_{\mathrm{out}}=\sum_{n=1}^{N}R_{n}P_{n}$$

where *R*_n_ is the weight (i.e., photoresponse) of the *n*th pixel, *P*_n_ is the input light power at the *n*th pixel, and *N* is the total number of pixels. This equation demonstrates that the ferroelectric photomemristor network can perform multiply–accumulate operations between the input image and the photoresponsivity matrix, which is the foundation of real‐time image recognition. Subsequently, the output is sent to a neuron unit composed of an amplifier circuit and an analog‐to‐digital converter (ADC), as shown in Figure 5d. The neuron unit converts the output current into a voltage signal, and through voltage comparison, this signal is further represented as one of four voltage levels. These voltage levels correspond to the recognition results of “left rear”, “forward”, “stop”, “backward”, and “right back”, respectively. Figure 5e illustrates the training flowchart, which mainly consists of two processes: inference and weight update. During inference, the traffic sign is projected onto the ferroelectric photomemristor network. The training objective is to minimize the difference between the network's recognition result and the true label of the traffic sign, a difference measured by the mean squared error cost Equation (4):

(4) $$L=\frac{1}{M}\sum_{i=1}^{M}{\left(y_{i}-{\hat{y}}_{i}\right)}^{2}$$

Here, *y*_i_ is the *V*_output_ of the *i*th input image after passing through the hyperbolic tangent activation function, *ŷ*_i_ is the true label of the nth input image, and *M* is the number of input images. To minimize this cost function, the weights must be updated to their optimal values. A gradient descent algorithm is employed to guide the weight update. In this algorithm, the target weights are calculated by Equation (5) in software:

(5) $$R_{n}:=R_{n}-\alpha \frac{\partial L}{\partial R_{n}}$$

where the “:=” symbol denotes assignment and *α* represents the learning rate. Next, using the BD‐CL programming scheme, the photoresponse of each device is experimentally adjusted to its corresponding target weight value. At this point, one training cycle is complete. The training can be automatically repeated over multiple cycles until the network converges. Figure 5f depicts the output currents of the ferroelectric photomemristor network for five traffic signs across different training epochs. At the initial epoch (Ep #0), all output currents fall within the range associated with the “stop” sign, indicating that the ferroelectric photomemristor network initially recognizes only the “stop” sign. As training progresses, an increasing number of traffic signs are correctly identified, with full 100% recognition accuracy achieved by Ep #10. Finally, the trained ferroelectric photomemristor network is tested using the same five traffic signs as those used during training. Figure 5g–k displays the vehicle navigation based on the real‐time recognition results of the trained network, and the vehicle responds correctly to all traffic signs.

## 3 Conclusions

This study proposes an in situ training of multifunctional signal processing in machine vision systems based on the strain engineering optimized ferroelectric photomemristors. The device exhibits excellent ferroelectric performance (*P*_r_ = 94 µC cm^−2^), which depends on the lattice matching between PZT and SRO. The device exhibits a significant photoelectric response, including an *I*_sc_ of 183.35 nA, enhancing the anti‐interference capability of the machine vision system. Additionally, photovoltaic testing of the device demonstrates reliable *I*_sc_ switching capability, achieving 20 000 s of light‐switching durability. It can adapt to light stimuli of varying power and wavelengths, making it suitable for complex light source environments, while achieving a stable and tunable photovoltaic response state. Moreover, the device can utilize photoconductive states of different directions and magnitudes to achieve pattern classification and in situ training in the machine vision system. The ferroelectric photomemristor network can not only enable the processing of static images but also achieve the in situ training of multifunctional signal processing for a mechanical car, which is beneficial for the development of an intelligent machine vision system based on ferroelectric photomemristor networks.

## 4 Experimental Methods

### 4.1 Device Fabrication

The chamber was evacuated to below 5 × 10^−4^ Pa; the temperature was raised to 700°C using a thermocouple, with the pressure adjusted to 3 Pa. Argon and oxygen were introduced at flow rates of 75 and 25 sccm, respectively, with a sputtering power of 50 W and a target‐to‐substrate distance of 54 mm, resulting in the deposition of a 60‐nm‐thick SRO film. After deposition, the chamber was backfilled with oxygen to 8 × 10^4^ Pa before cooling to room temperature, obtaining SRO/STO, LNO/STO, and LSCO/STO heterostructures. For epitaxial PZT ferroelectric thin film fabrication via the sol‐gel technique: A plate furnace was preheated to 350°C. Precrystallized SRO/STO, LNO/STO, and LSCO/STO substrates were spin‐coated with PZT colloid using a two‐step process ‐ 500 rpm for 8 s followed by 4000 rpm for 42 s, forming wet PZT films. Films were baked at 350°C for 5 min in the plate furnace to remove organic residues. Subsequent annealing was performed in an oxygen atmosphere at 550°C for 1 h using a slow‐cooling protocol, yielding 130 nm‐thick epitaxial PZT films. To minimize work function mismatch effects on ferroelectric properties, symmetric Pt top electrodes (Pt/SRO, Pt/LNO, Pt/LSCO) were fabricated using shadow mask technology: Substrates were mounted on masks and loaded into a sputtering system. Pt deposition parameters: Base pressure <2 × 10^−4^ Pa, working pressure 3 Pa, 50 sccm Ar flow, 53 mm target distance, 60 W RF power. Process included 5 min presputtering for target cleaning and 130 s main sputtering, producing 35 nm Pt electrodes. Final post‐annealing at 550°C for 1 h in flowing oxygen ensured electrode crystallization.

### 4.2 Raman Testing

The Raman measurements were performed at room temperature using an HR Evolution 800 spectrometer (Horiba Jobin Yvon GmbH) equipped with a HeCd laser (633 nm, ≈3.815 eV). The excitation energy was selected to be greater than the bandgap of PZT (≈3.600 eV) [1, 2] to confine the laser penetration depth to a few nanometers beneath the film surface, thereby ensuring the stress analysis is specific to the PZT layer. The laser spot diameter on the samples was ≈3 µm, and the power was maintained below 1 mW to ensure a low energy density, thus safely neglecting any heating effects. The residual stress in the films was calculated from the phonon mode shift using the relation: *ω*(σ) = ω(0) – σ(∂ω/∂σ). Here, ω(0) = 346.5 cm^−1^ is the stress‐free frequency of the A_1_(2TO) mode, and the coefficient ∂ω/∂σ was estimated to be 13.9 cm^−1^ GPa^−1^. The stress σ was then determined from the measured peak shift.

### 4.3 Photovoltaic Performance Characterization

The photovoltaic performance was measured using a probe station equipped with a Keysight B1500A analyzer. All devices feature a circular Pt top electrode with a diameter of 100 µm and a geometric area of 7.85 × 10^−5^ cm^2^. During measurement, a 405 nm continuous‐wave laser at 100 mW cm^−2^ was focused to a 100 µm diameter spot, ensuring it fully covered the top electrode so that all photogenerated carriers originate from the defined electrode region.

### 4.4 Ferroelectric Photomemristor Network Measurements

The setup for the in situ training of the ferroelectric photomemristor network mainly includes a microcontroller unit (MCU), 8‐channel 16‐bit ADC, 12‐bit digital‐to‐analog converters (DACs), trans‐impedance amplifiers (TIAs), and a personal computer (PC). The MCU used in our experiment was STM32. When performing the training, the ferroelectric photomemristor network produced a photocurrent upon the application of illumination. The current signal output by the array first enters the signal conversion module composed of an amplifier and TIAs, which were used to convert currents to voltages and amplify the output voltage signals. Then converted voltage signal entered an analogue‐to‐digital converter controlled by the MCU, which was responsible for reading voltage signals, respectively. The PC implemented the gradient descent algorithm, and relayed the result to the MCU. The PC communicated with STM32 through Universal Asynchronous Transceiver to obtain data. The MCU then instructed the DACs to apply pulses to the ferroelectric photomemristor network to update the weights. The training proceeded automatically for multiple epochs. The ferroelectric photomemristor network trained was used to perform the real‐time image recognition for a prototype autonomous vehicle. The vehicle also comprised a motor system. The real‐time recognition result of the ferroelectric photomemristor network was directly sent to the motor system of the vehicle to control its movements.

## Conflicts of Interest

The authors declare no conflicts of interest.

## Supporting information




**Supporting File**: advs73513‐sup‐0001‐SuppMat.docx

## Data Availability

The data that support the findings of this study are available from the corresponding author upon reasonable request.
