# Supplementary material for: Giant Switchable Remanent Polarization and Photocurrent in Ferroelectric Thin Film Photomemristor for In Situ Training
Source: Adv Sci (Weinh). 2026 Feb 27;13(20):e17077. doi: 10.1002/advs.202517077 (PMC13067828; doi:10.1002/advs.202517077)
Supplement: Supplementary file 1 — Supporting File: advs73513‐sup‐0001‐SuppMat.docx [file ADVS-13-e17077-s001.docx]

Supporting Information

**Giant switchable Remanent Polarization and Photocurrent in Ferroelectric Thin Film photomemristor for In Situ Training**

*Zhen Zhao^1, #^, Chengze Sun^2, #^, Zhijin Duo^1^, Yue Hou^1^, Zhanfeng Wang^1^, Jikang Xu^2^, Ziye Li^2^, Fu Wang^2^, Pengfei Li^2^, Ying Liu^2^, Yongqing Jia^2^, Kangbo Zhao^2^, Jia Wu^2^, Biao Yang^2^, Weifeng Zhang^2^, Weidong Sun^2^, Jiacheng Wang^2^, Jinxia Liu^2^, Junfeng Yu^2^, Xiang Ying^2^, Jianxin Guo^1^, Xiaobing Yan^1, 2, *^*

^1^ Key Laboratory of Optic-Electronic Information Materials of Hebei Province, School of Life Sciences, Institute of Life Science and Green Development, Key Laboratory of Brain-Like Neuromorphic Devices and Systems of Hebei Province, College of Physics Science and Technology, Hebei University, Baoding, 071002, China.

^2^ College of Electronic and Information Engineering. Hebei University, Baoding, 071002, China.

^#^ Zhen Zhao, Chengze Sun contributed equally to this work.

^*^ Corresponding Authors: E-mail addresses: [yanxiaobing@ime.ac.cn](mailto:yanxiaobing@ime.ac.cn) (Xiaobing Yan)


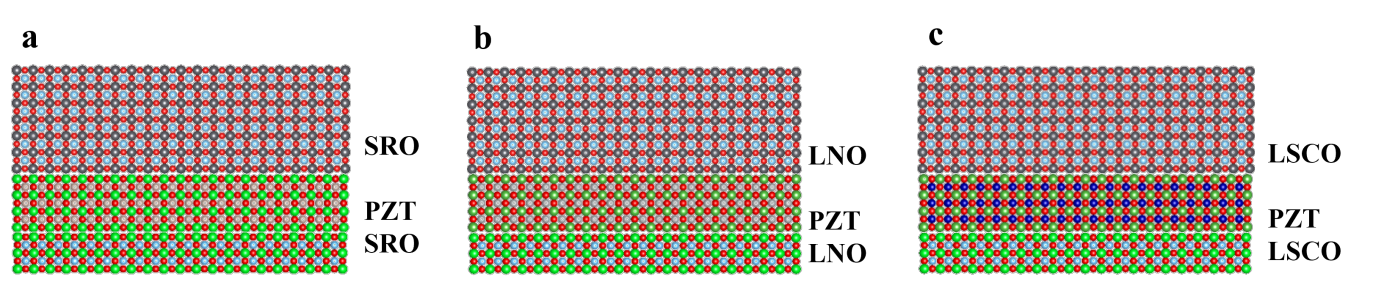


**Figure S1.** Three distinct heterostructures. a) Structure Ⅰ: SRO/PZT/SRO/STO. b) Structure Ⅱ: LNO/PZT/LNO/STO. c) Structure Ⅲ: LSCO/PZT/LSCO/STO.

This study compares heterostructures with three different electrodes, all of which are deposited on STO substrates and epitaxially grown with PZT ferroelectric layers. The difference between the three structures lies in the electrode materials, which are: Structure Ⅰ (Figure S1a): SRO/PZT/SRO/STO, Structure Ⅱ (Figure S1b): LNO/PZT/LNO/STO, and Structure Ⅲ (Figure S1c): LSCO/PZT/LSCO/STO. This design aims to maintain the substrate (STO) and ferroelectric layer (PZT) unchanged, and explore the role of electrode interface in ferroelectric properties and photoresponse by systematically changing the electrode material.

The core purpose of choosing this comparative scheme is to investigate the influence of different electrode materials on the ferroelectric layer. According to the comprehensive research results, the compressive stress applied by the SRO electrode to the PZT ferroelectric layer is beneficial for polarization reversal and efficient carrier transport, thus explaining its superior comprehensive performance.


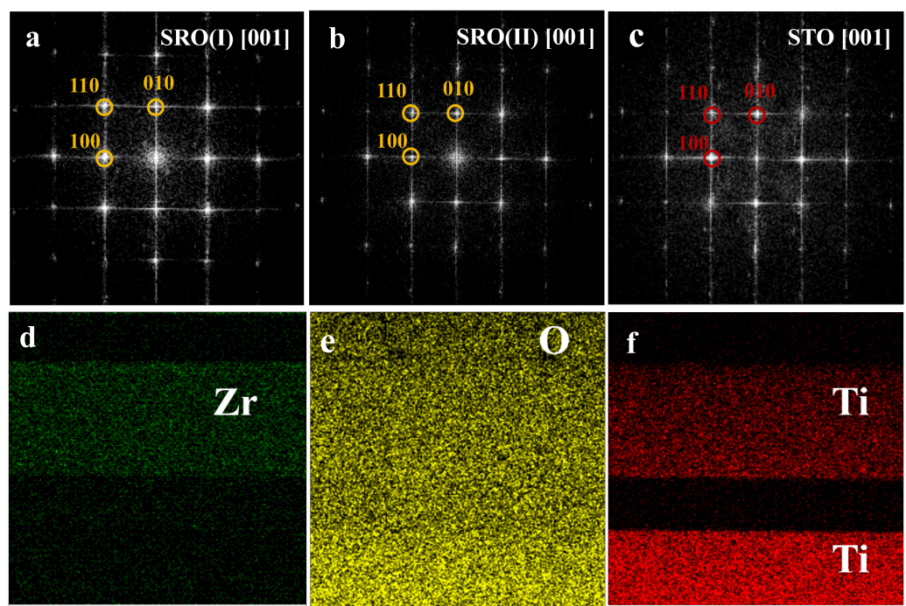


**Figure S2.** Additional TEM characterization. a) FFT pattern of the SRO(I) layer region. b) FFT pattern of the SRO(II) layer region.c) FFT pattern of the STO layer region. d) Element mapping of Zr. e) Element mapping of O. f) Elemental mapping of Ti.


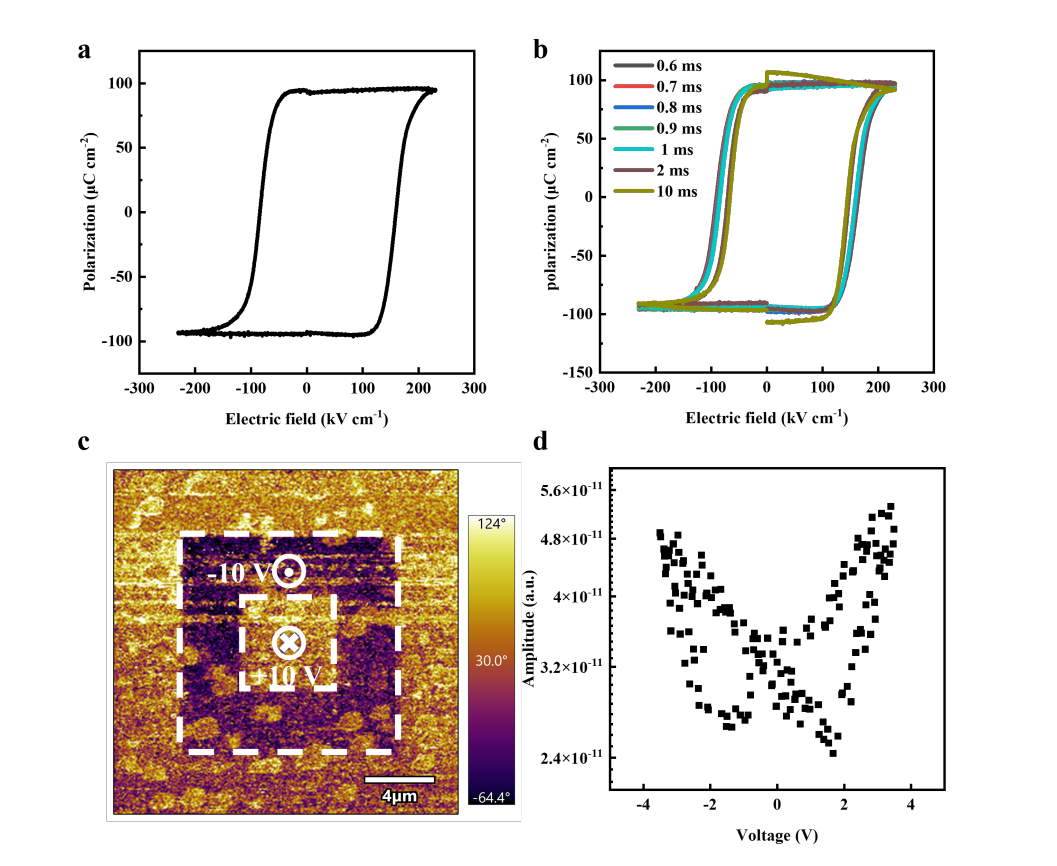


**Figure S3.** Further explore the ferroelectricity of structure Ⅰ. a) *P-E* measurement using the PUND pulse method. b) Frequency dependent *P-E* hysteresis loops. c) PFM phase domain diagram. d) Amplitude hysteresis loop.


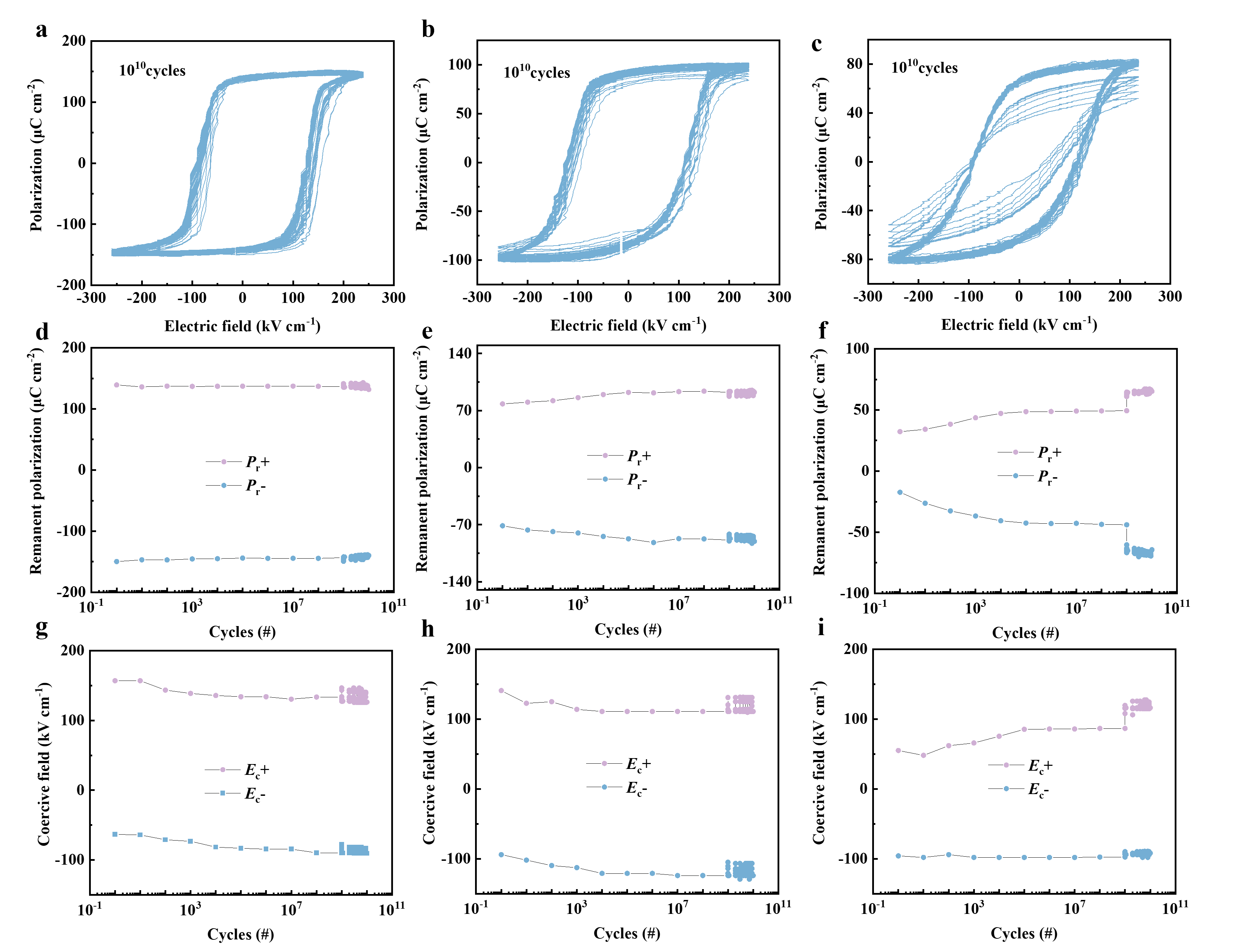


**Figure S4.** Fatigue resistance characterization of the different bottom electrode devices. *P-E* hysteresis loops (a), statistical analysis of *P*_r_ (d), and *E*_c_ evolution (g) after 10^10^ switching cycles of the Pt/SRO/PZT/SRO/STO device. *P-E* hysteresis loops (b), statistical analysis of *P*_r_ (e), and *E*_c_ evolution (h) after 10^10^ switching cycles of the Pt/LNO/PZT/LNO/STO device. *P-E* hysteresis loops (c), statistical analysis of *P*_r_ (f), and *E*_c_ evolution (i) after 10^10^ switching cycles of the Pt/LSCO/PZT/LSCO/STO device.


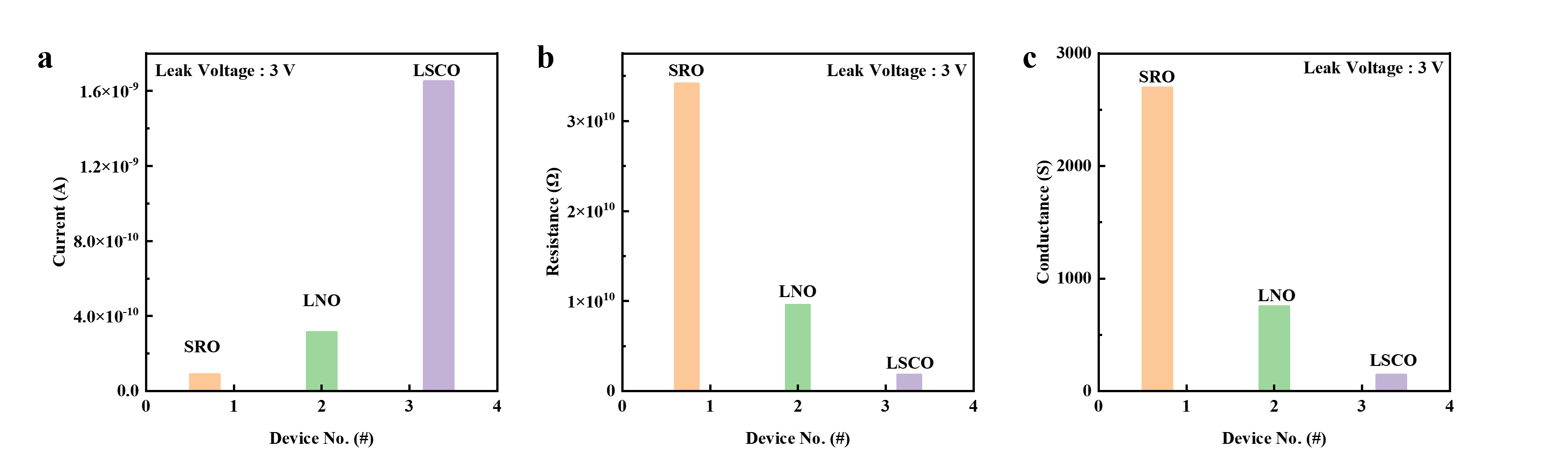


**Figure S5.** Leakage current characterization of devices with different bottom electrodes. Leakage current comparison (a), resistance comparison (b), and resistivity comparison (c) of three bottom electrode-based devices under a 3 V write pulse.


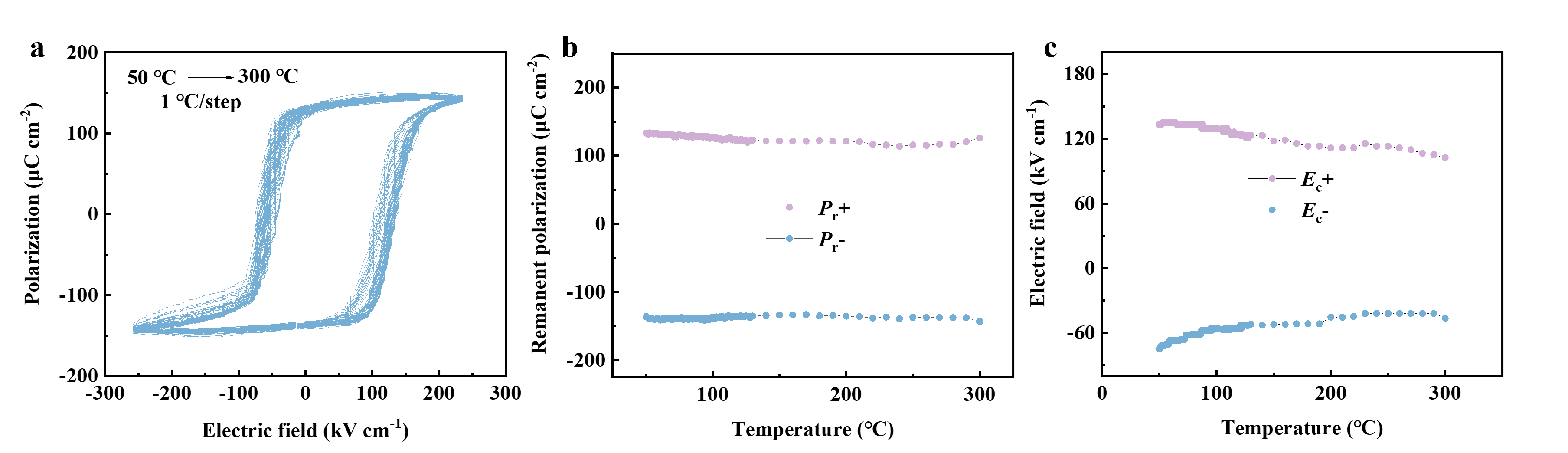


**Figure S6.** Temperature-dependent ferroelectric polarization characterization of the devices. *P-E* hysteresis loops at different temperatures (a), *P*_r_ variation (b), and *E*_c_ evolution (c) of the devices.


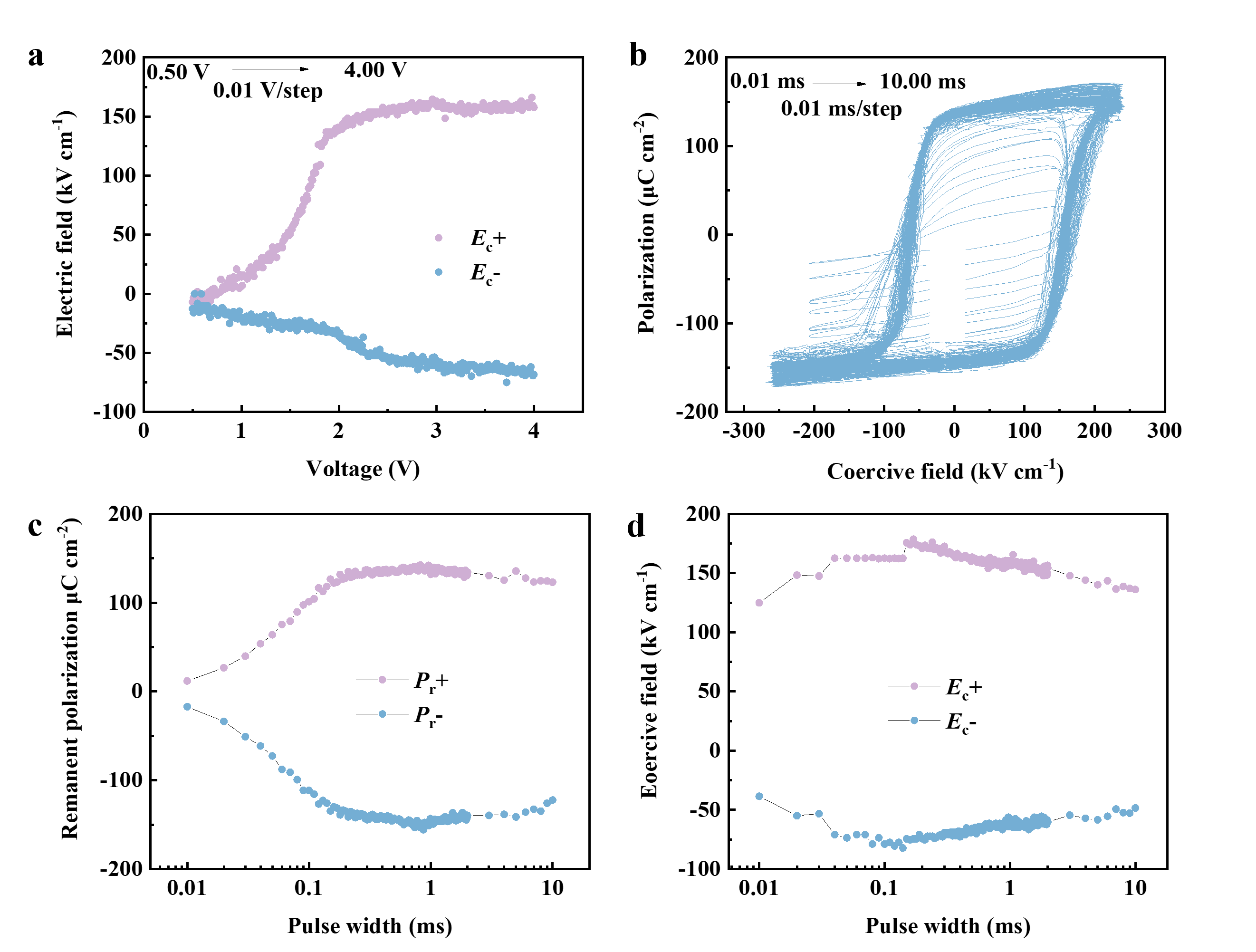


**Figure S7.** Multilevel resistive state characterization of the devices. a) *E*_c_ evolution under different write voltage pulses. *P-E* hysteresis loops (b), *P*_r_ (c), and *E*_c_ (d) of devices after writing with pulses of different widths.


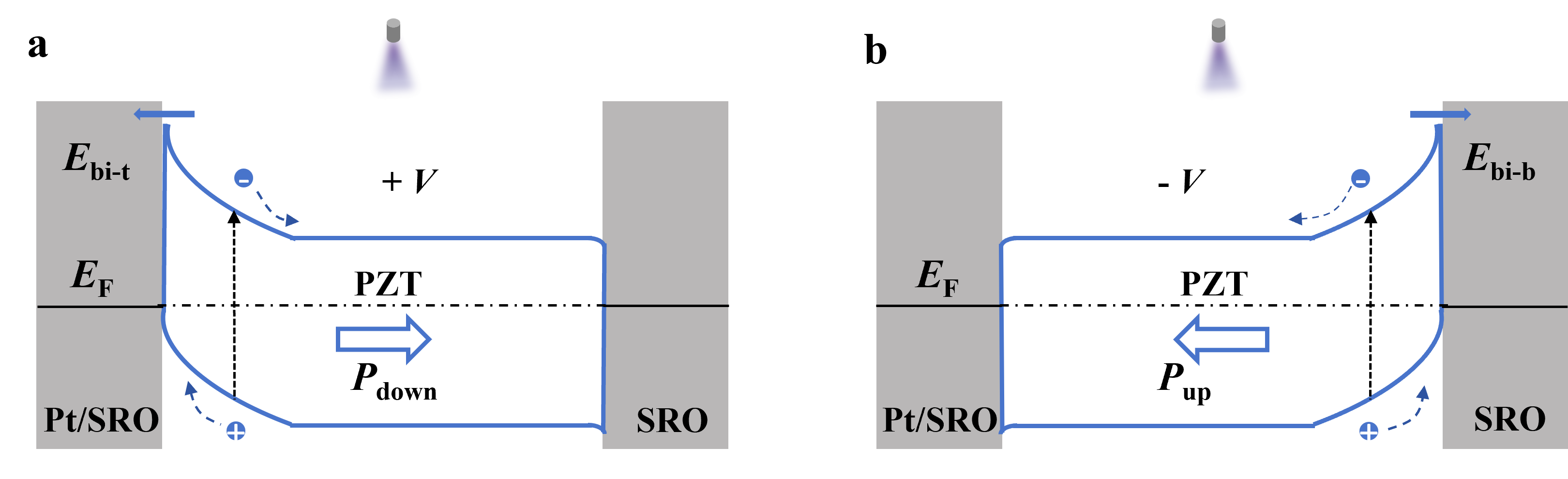


**Figure S8.** Schematic energy band diagrams of the device in the complete *P*_down_ (a) and *P*_down_ (b) states.

Previous studies have established that polarization-modulated Schottky barriers are responsible for the switchable photovoltaic effect in Pt/PZT/SRO devices^[1,2]^. In our study, the epitaxial PZT film, being an n-type semiconductor^[3]^, forms Schottky barriers with both the top Pt and bottom SRO electrodes. In a hypothetical non-polarized PZT state, the potential barrier heights at the top (Pt/PZT) and bottom (PZT/SRO) interfaces would be similar (~5.2 eV). However, ferroelectric polarization significantly alters these barrier heights and their associated built-in fields (*E*_bi-t_ and *E*_bi-b_). In the fully *P*_down_ state, the positive polarization charge at the top interface reduces the barrier, making *E*_bi-t_ dominant and generating a net negative photocurrent (Figure S8a). Conversely, in the fully *P*_up_ state, the negative polarization charge at the bottom interface increases that barrier, enhancing *E*_bi-b_, while the positive charge at the top interface reduces or even eliminates *E*_bi-t_ (Figure S8b)^[4,5]^. Consequently, the downward *E*_bi-b_ dominates, producing a net positive photocurrent. In intermediate polarization states, the relative proportion of upward and downward domains dictates the magnitude and direction of the net photocurrent, enabling multi-level photocurrent states. Therefore, the model of polarization-modulated Schottky barriers effectively explains the polarization-controlled switchable photoresponse in our Pt/SRO/PZT/SRO/STO device.


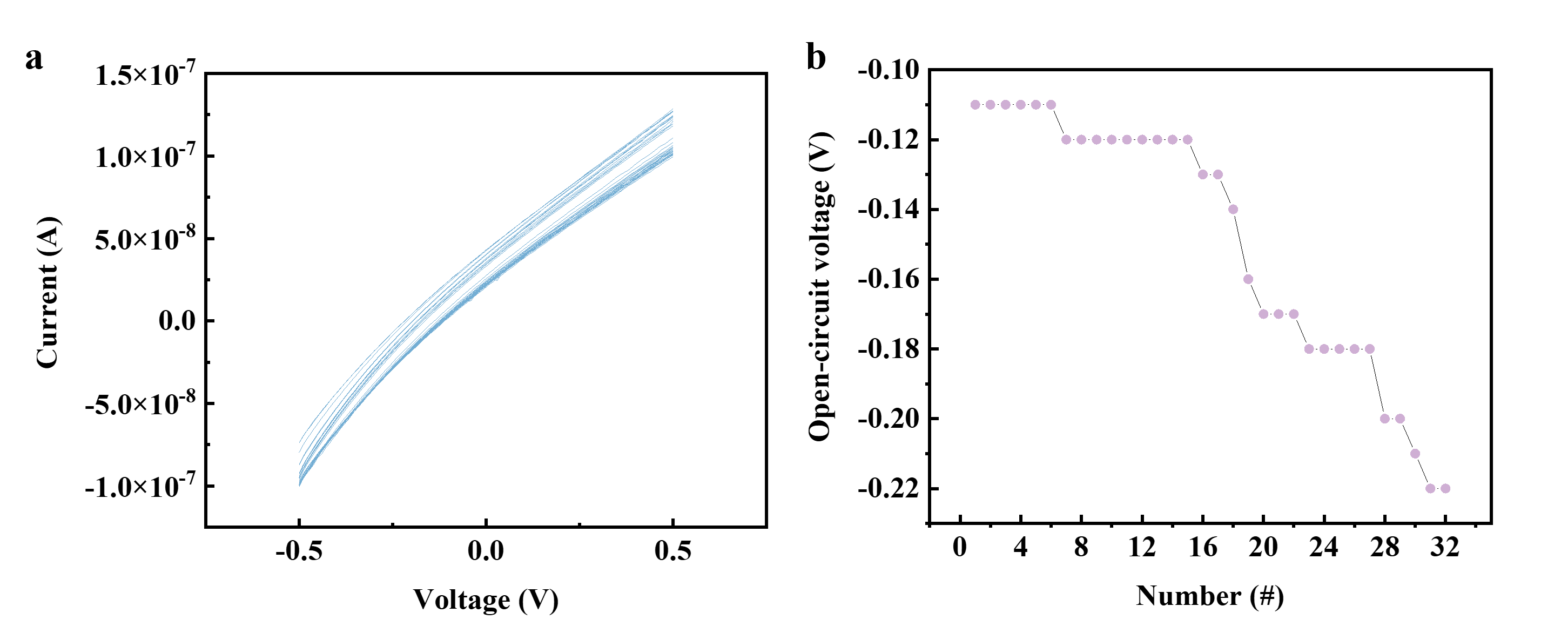


**Figure S9.** Characterization of polarization state-dependent device behavior. a) *I–V* curves of the device in *P*_down_ state. b) Statistical analysis of *V*_oc_ variation in *P*_down_ state.


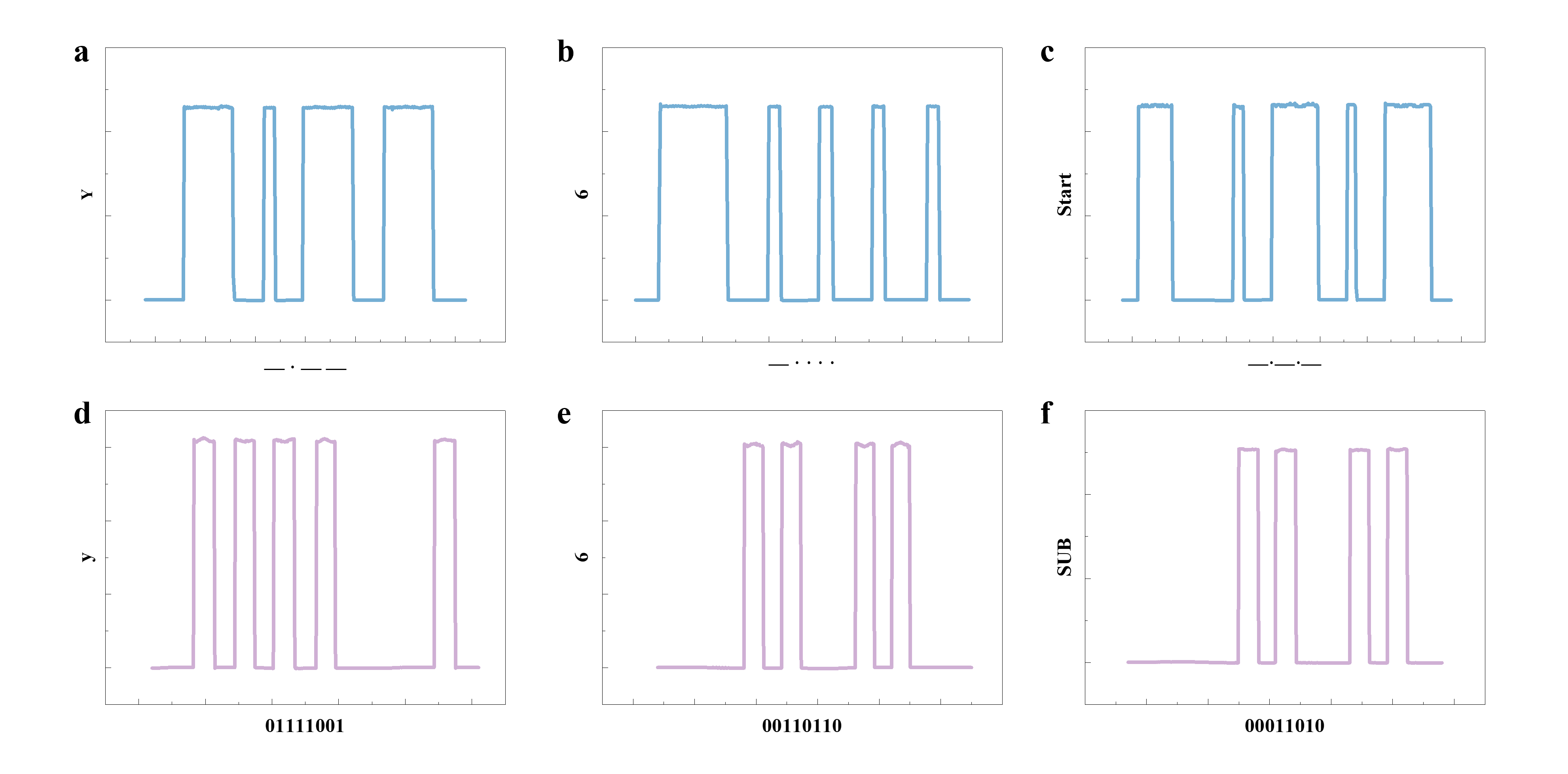


**Figure S10.** The device emulates Morse code characters (a–c) and ASCII numerical digits (d–f) via light pulses.


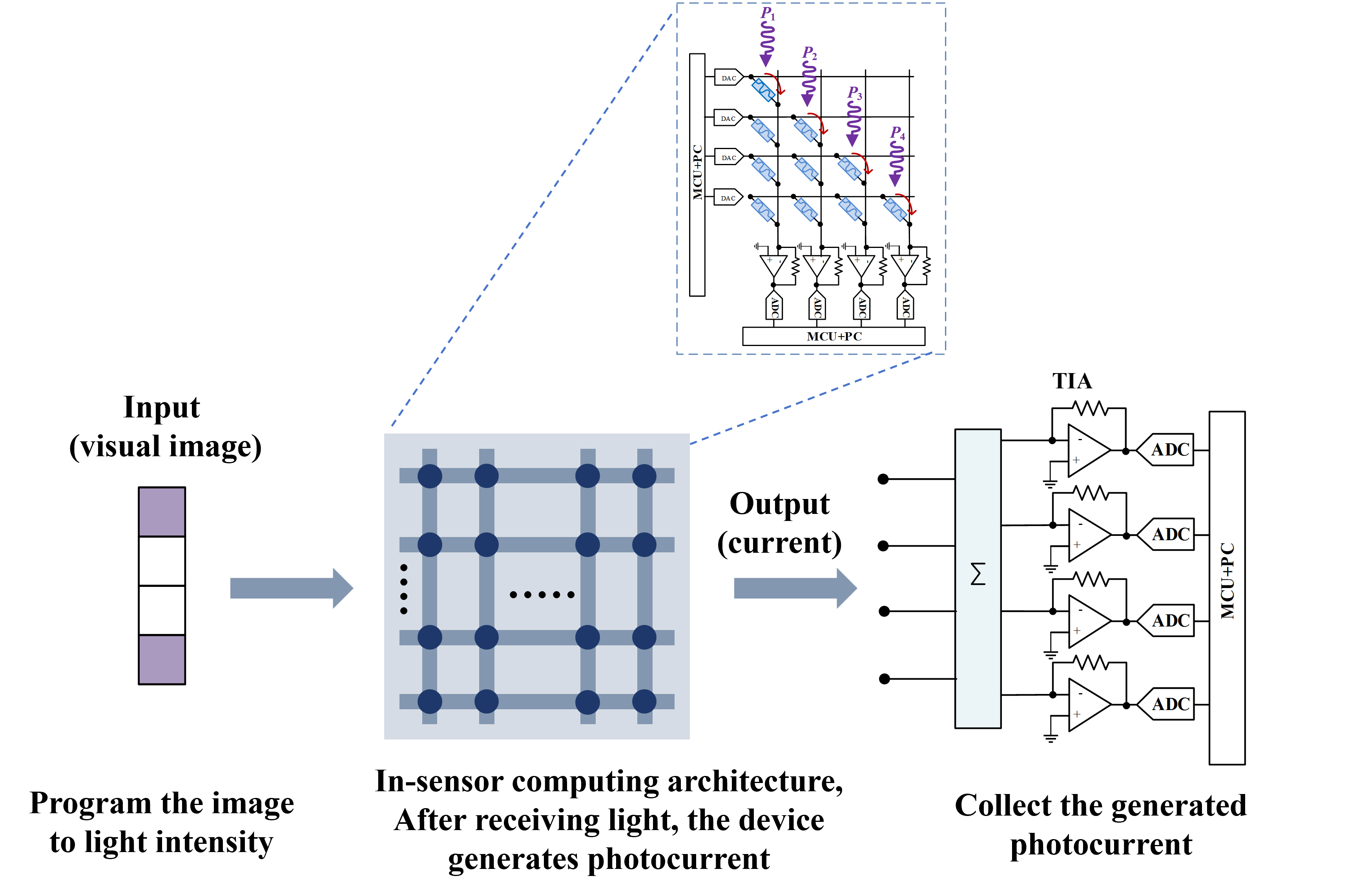


**Figure S11.** Flowchart of device implementation instructions.

Each photomemristor converts incident light into a photocurrent according to its optical sensitivity, which effectively acts as its weight. The photomemristors are connected in parallel, and their output currents are summed at a common node. The total current thus represents the combined optical response of the entire array.

This summed current is then amplified and converted into a voltage signal using a transimpedance amplifier. The voltage output is digitized and classified into five discrete levels, each corresponding to a specific driving command. In this way, the memristor array and readout circuit jointly perform the multiply-and-accumulate operation directly within the sensing layer achieving image recognition without external computation. The signal flow (Figure S11): Starting from a laser light pattern, proceeding through optical alignment, a ferroelectric photomemristor array, a current summation node, an amplifier, and an analog-to-digital converter (or comparator), and finally reaching the control unit.

**Table S1.** Comparison of *P*_r_ and *E*_c_ in PZT-based ferroelectric devices reported in the literature.

| No. | Device materials | Remanent  Polarization  [μC cm^-^²] | Coercive  Electric Field  [kv cm^-1^] | Ref. |
| --- | --- | --- | --- | --- |
| 1 | PZT/SRO/STO | 60 | 100 | [Nature Communications](https://www.nature.com/ncomms). 2025, 16, 3192 |
| 2 | Co/Alq_3_/PZT/LSMO | 1.5 | 4000 | [Nature Communications](https://www.nature.com/ncomms). 2014, 5, 4693 |
| 3 | PZT/Nb-STO | 80 | 600 | [Nature Communications](https://www.nature.com/ncomms). 2014, 5, 4693 |
| 4 | PZT/SiO_2_/Si | 75 | 50 | [Nature Communications](https://www.nature.com/ncomms). 2025, 16, 6340 |
| 5 | PZT/SRO/STO | 45.9 | 500 | [Nature Communications](https://www.nature.com/ncomms). 2025, 16, 3882 |
| 6 | PZT/SRO/STO | 80 | 142 | [Nature Communications](https://www.nature.com/ncomms). 2025, 16, 421 |
| 7 | PZT/SRO/STO | 80 | 205 | [Nature Communications](https://www.nature.com/ncomms). 2022, 13, 1707 |
| 8 | PZT/Metglas | 20 | 125 | Adv.Mater. 2023, 35, 32, 2303553 |
| 9 | PZT/LSMO/LAO | 82 | 1.4 | Adv. Funct. Mater. 2024, 34, 45, 2406666 |
| 10 | PZT/PET | 43 | 20 | Adv. Funct. Mater. 2025, 35, 4, 2414211 |
| 11 | PZT/LNO/ZRC | 40.2 | 40.3 | Adv. Funct. Mater. 2022, 32, 52, 2209297 |
| 12 | LNO/PZT/LNO/STO | 38 | 20 | Adv. Funct. Mater. 2020, 30, 52, 2005397 |
| 13 | ZnO NW/PZT/Pt/SiO_2_/Si | 20 | 100 | ACS Nano. 2009, 3, 3, 700-706 |
| 14 | PZT/LNO/SUS430 | 79 | 42 | ACS Appl. Electron. Mater. 2024, 6, 9, 6384-6390 |
| 15 | PZT/SRO/STO | 45.4 | 175 | ACS Appl. Electron. Mater. 2024, 6, 2, 1063-1070 |
| 16 | PZT/SRO/STO | 60 | 300 | Nano Lett. 2024, 24, 40, 12426-12432 |
| 17 | PZT/Mica | 10 | 50 | [Nano Energy](https://www.sciencedirect.com/journal/nano-energy). 2021, 85, 105984 |
| 18 | PZT/PSS/Ag | 56 | 80 | Advanced Science. 2024, 11, 31, 2400174 |
| 19 | PZT/PVDF&CNTs | 17 | 1.1 | Journal of Materiomics. 2024, 1063- 1070 |
| 20 | xPb(Nb_2/3_Ni_1/3_)O_3_-(1-x)Pb(Zr_0.3_Ti_0.7_)O_3_ | 62 | 9.1 | Journal of Materials Science & Technology. 2024, 37- 43 |
| 21 | Pb_1-x_La_x_(Zr_0.52_Ti_0.48_)O_3_ | 31 | 15.5 | Chemical Engineering Journal. 2024, 152004 |
| 22 | PSZT-Fe | 30 | 1.8 | Journal of Advanced Ceramics. 2021, 10, 3, 587- 595 |
| 23 | PbZr_1-x_Ti_x_ O_3_  (Pb:Zr:Ti=110:52:48) | 10 | 140 | Nano Energy. 2021,105984 |
| 24 | PbZrO_3_ | 2.3 | 40 | Adv.Mater. 2023, 35, 2206541 |
| 25 | PNN-PZT-x%LN | 50 | 9 | Journal of Materiomics. 2024, 995-1003 |
| 26 | PCZT | 30 | 500 | Journal of Materials Science & Technology. 2024, 171, 139- 146 |
| 27 | PZT | 34.4 | 12 | Additive Manufacturing. 2023, 78, 103857 |
| 28 | 0.2PCN-0.8PZT | 40.4 | 16 | Adv.Energy Mater. 2023, 13, 2301796 |
| 29 | PIMN-34PT | 35 | 575 | Journal of Materials Science & Technology. 104.  2022, 104, 119- 126 |
| 30 | PLZT | 60 | 300 | ACS Appl. Mater. Interfaces. 2020, 12, 48, 53957-53965 |
| 31 | PZT | 70 | 75 | Adv. Electron. Mater. 2022, 8, 2100612 |
| 32 | GaN/PZT/ITO | 41.6 | 500 | Adv. Electron. Mater. 2024, 10, 2300588 |
| 33 | CBO/G-Au/PZT | 26 | 900 | Adv. Funct. Mater. 2024, 34, 2316409 |
| 34 | SRO/PZT/SRO/STO | 94 | 120 | This work |

**Table S2.** Comparison of *J*_sc_ (*I*_sc_/*S*) in ferroelectric photovoltaic devices reported in the literature.

| No. | Device structure | *J*_sc_ (μA cm^-2^) | Ref. |
| --- | --- | --- | --- |
| 1 | PZT/SRO/STO | 34 | [Nature Communications](https://www.nature.com/ncomms). 2022, 13, 1707 |
| 2 | PZT/SRO/STO | 48 | [Nature Communications](https://www.nature.com/ncomms). 2025, 16, 2341 |
| 3 | (iso-pentylammonium)_2_(ethylammonium)_2_Pb_3_I_10_/Au | 1.5 | [Nature Communications](https://www.nature.com/ncomms). 2021, 12, 284 |
| 4 | ReSe_2_/Au | 44 | [Nature Communications](https://www.nature.com/ncomms). 2025, 16, 6313 |
| 5 | BiSmFe_2_O_6_/Nb-SrTiO_3_ | 13.03 | Science Advances. 2025, 11, 17. |
| 6 | (STO)_x_/(BTO)_y_/(CTO)_z_/Nb:STO | 10 | Science Advances. 2021, 7, 23 |
| 7 | BFO/STO | 179 | Adv. Mater. 2025, 37, 13, 2420333 |
| 8 | ԑ-InSe/Graphene/SiO_2_/Si | 1222 | Adv. Mater. 2024, 36, 44, 2410696 |
| 9 | EA_4_Pb_3_Br_10_/PS | 0.08 | Adv. Mater. 2021, 33, 32, 2101263 |
| 10 | BZT-BCT-xT/Au | 0.2625 | Adv. Mater. 2025, 37, 9, 2418023 |
| 11 | EA_4_Pb_3_Cl_1_0_4_/Ag | 0.00063 | J. Am. Chem. Soc. 2020, 142, 1, 55-59 |
| 12 | (CPA)_2_/FAPb_2_Br_7_/Ag | 0.134 | J. Am. Chem. Soc. 2022, 144, 31, 14031-14036 |
| 13 | (3-bromopropylaminium)_2_(formamidinium)Pb_2_Br_7_/Au | 140 | J. Am. Chem. Soc. 2021, 143, 20, 7593-7598 |
| 14 | BBFMO/NSTO | 42.23 | J. Am. Chem. Soc. 2024, 146, 20, 13934-13948 |
| 15 | PZT/LSMO/LAO | 961 | Adv. Funct. Mater. 2024, 34, 45, 2406666 |
| 16 | PZT/SRO/STO | 40 | Adv. Electron. Mater. 2022, 8, 2100612 |
| 17 | 0.88(Na_0.5_Bi_0.5_TiO_3_)-0.12(Ba_1-1.5x_Sm_x_TiO_3_) | 4 | Journal of Materiomics 10 (2024) 975-983 |
| 18 | ADPVE/MoSe_2_/LiNbO_3_ | 1.5 | Nano Letters. 24,33(2024) |
| 19 | CsPbBr_3_ | 0.075 | Adv. Mater. 2024, 2403875 |
| 20 | Cd_S_b_2_Se_3_Br_2_ | 0.1 | Adv. Mater. 2024, 2406984 |
| 21 | CIPS | 0.016 | Nano Energy 129 (2024) 110068 |
| 22 | ReS_2_ | 1.75 | Nature Communications (2024) 15:501 |
| 23 | CsPbBr_3_/Glass | 0.19 | ACS Nano 2024, 18, 23310−23319 |
| 24 | SBFO/Pt | 31.4 | ACS Materials Lett. 2024, 6, 4248−4254 |
| 25 | SRO/PZT/SRO/STO | 1878±144 | This work |

References:

[1] Tan, Zhengwei, et al. “Thinning ferroelectric films for high-efficiency photovoltaics based on the Schottky barrier effect.” *NPG Asia Materials* 11.1 (2019): 20.

[2] Cui, Boyuan, et al. “Ferroelectric photosensor network: an advanced hardware solution to real-time machine vision.” *Nature Communications* 13.1 (2022): 1707.

[3] Pintilie, L., et al. “Ferroelectric polarization-leakage current relation in high quality epitaxial Pb(Zr,Ti)O_3_ films.” *Physical Review B—Condensed Matter and Materials Physics* 75.10 (2007): 104103.

[4] Lee, Daesu, et al. “Polarity control of carrier injection at ferroelectric/metal interfaces for electrically switchable diode and photovoltaic effects.” *Physical Review B—Condensed Matter and Materials Physics* 84.12 (2011): 125305.

[5] Fang, Liang, et al. “Switchable photovoltaic response from polarization modulated interfaces in BiFeO_3_ thin films.” *Applied Physics Letters* 104.14 (2014).
